# Association between doubly labelled water-calibrated energy intake and objectively measured physical activity with mortality risk in older adults

**DOI:** 10.1186/s12966-023-01550-x

**Published:** 2023-12-25

**Authors:** Daiki Watanabe, Tsukasa Yoshida, Yuya Watanabe, Yosuke Yamada, Motohiko Miyachi, Misaka Kimura

**Affiliations:** 1 Faculty of Sport Sciences, Waseda University, 2-579-15 Mikajima, Tokorozawa-city, Saitama 359-1192 Japan; 2 National Institute of Health and Nutrition, National Institutes of Biomedical Innovation, Health and Nutrition, 17-34 Senrioka-Shimmachi, Settsu-city, Osaka 566-0002 Japan; 3 Institute for Active Health, Kyoto University of Advanced Science, 1-1 Nanjo Otani, Sogabe-cho, Kameoka-city, Kyoto, 621-8555 Japan; 4 Senior Citizen’s Welfare Section, Kameoka City Government, 8 Nonogami, Yasu-machi, Kameoka-city, Kyoto, 621-8501 Japan; 5 Faculty of Sport Study, Biwako Seikei Sport College, 1204 Kitahira, Otsu-city, Shiga 520-0503 Japan; 6 Laboratory of Applied Health Sciences, Kyoto Prefectural University of Medicine, 465 Kajii-cho, Kamigyo-ku, Kyoto-city, Kyoto, 602-8566 Japan

**Keywords:** Energy intake, Physical activity, Recovery biomarker, Interaction, Dose–response relationship

## Abstract

**Background:**

Physical activity or biomarker-calibrated energy intake (EI) alone is associated with mortality in older adults; the interaction relationship between the combined use of both factors and mortality has not been examined. We evaluated the relationship between mortality and calibrated EI and step counts in older adults.

**Methods:**

This prospective study included 4,159 adults aged ≥65 years who participated in the Kyoto-Kameoka study in Japan and wore a triaxial accelerometer between 1 April and 15 November 2013. The calibrated EI was calculated based on a previously developed equation using EI biomarkers. The step count was obtained from the accelerometer ≥ 4 days. Participants were classified into the following four groups: low EI (LEI)/low step counts (LSC) group (EI: <2,400 kcal/day in men and <1,900 kcal/day in women; steps: <5,000 /day), *n* = 1,352; high EI (HEI)/LSC group (EI: ≥2,400 kcal/day in men and ≥1,900 kcal/day in women; steps: <5,000 /day), *n* = 1,586; LEI/high step counts (HSC) group (EI: <2,400 kcal/day in men and < 1,900 kcal/day in women; steps: ≥5,000 /day), *n* = 471; and HEI/HSC group (EI: ≥2,400 kcal/day in men and ≥1,900 kcal/day in women; steps: ≥5,000 /day), *n* = 750. Mortality-related data were collected until 30 November 2016. We performed a multivariable Cox proportional hazard analysis.

**Results:**

The median follow-up period was 3.38 years (14,046 person-years), and 111 mortalities were recorded. After adjusting for confounders, the HEI/HSC group had the lowest all-cause mortality rate compared to other groups (LEI/LSC: reference; HEI/LSC: hazard ratio [HR]: 0.71, 95% confidence interval [CI]: 0.41–1.23; LEI/HSC: HR: 0.59, 95% CI: 0.29–1.19; and HEI/HSC: HR: 0.10, 95% CI: 0.01–0.76). No significant interaction was observed between the calibrated EI and steps with mortality. The spline model showed that 35–42 kcal/100 steps/day of EI/100 steps was associated with the lowest mortality risk.

**Conclusions:**

HR mortality risk was lowest at 35–42 kcal/100 steps/day, suggesting that very high (≥56 kcal) or low (<28 kcal) EI/100 steps are not inversely associated with mortality. Adherence to optimal EI and adequate physical activity may provide sufficient energy balance to explain the inverse association with mortality among older Japanese adults.

**Supplementary Information:**

The online version contains supplementary material available at 10.1186/s12966-023-01550-x.

## Background

Diet and physical activity are major modifiable risk factors related to longevity worldwide [1]. Both energy intake (EI) and physical activity contribute to the dynamic equilibrium of energy balance, and body mass reflects long-term changes in this balance [2]. In older adults, body mass is closely associated with mortality risk [3]. Therefore, an evidence-based approach that considers both EI and physical activity status is important when delivering lifestyle advice to older adults.

Self-reported data from questionnaires are prone to systematic errors associated with participant characteristics [4, 5], making accurate evaluation of EI [6–8] and physical activity level [7] challenging. Therefore, the use of biomarkers is advised for evaluating dietary intake in nutritional epidemiology studies [9]. Previous research has shown that biomarker-calibrated EI has a stronger association with risks of mortality [6] and cardiovascular disease [8] than uncalibrated EI. Similar findings have been reported for physical activity and cancer risk [7], with objectively evaluated physical activity using wearable devices showing a stronger association with health outcomes than self-reported measures [10]. The relationship between mortality risk and calibrated EI and objectively evaluated physical activity should be assessed to minimise systematic errors.

The analysis of the international doubly labelled water (DLW) method database indicated that total energy expenditure (TEE) decreases in older men and women starting from age 60 [11]. Therefore, role of physical activity and EI should be understood in relation to the mortality risk of older adults who experience changes in energy balance. The daily step counts are a simple, easily understandable, and objective measure of physical activity [12] that is effective for setting physical activity goals and motivating increased physical activity [13]. Furthermore, step counts can be evaluated using versatile tools, including smartphones and wearable devices. However, to the best of our knowledge, the relationship between step counts, calibrated EI, and mortality risk has not been examined to date. Therefore, this study aimed to investigate the relationship between the combination of calibrated EI and step counts and all-cause mortality risk among a community-based longitudinal cohort study on older adults. Previous studies have shown that diet and physical activity are independent risk factors for total mortality [1, 7]; thus, we hypothesised that individuals with high EI (HEI) and high step counts (HSC) would have the strongest inverse relationship with all-cause mortality risk.

## Methods

### Study population and baseline characteristic assessment

**Fig. 1:**
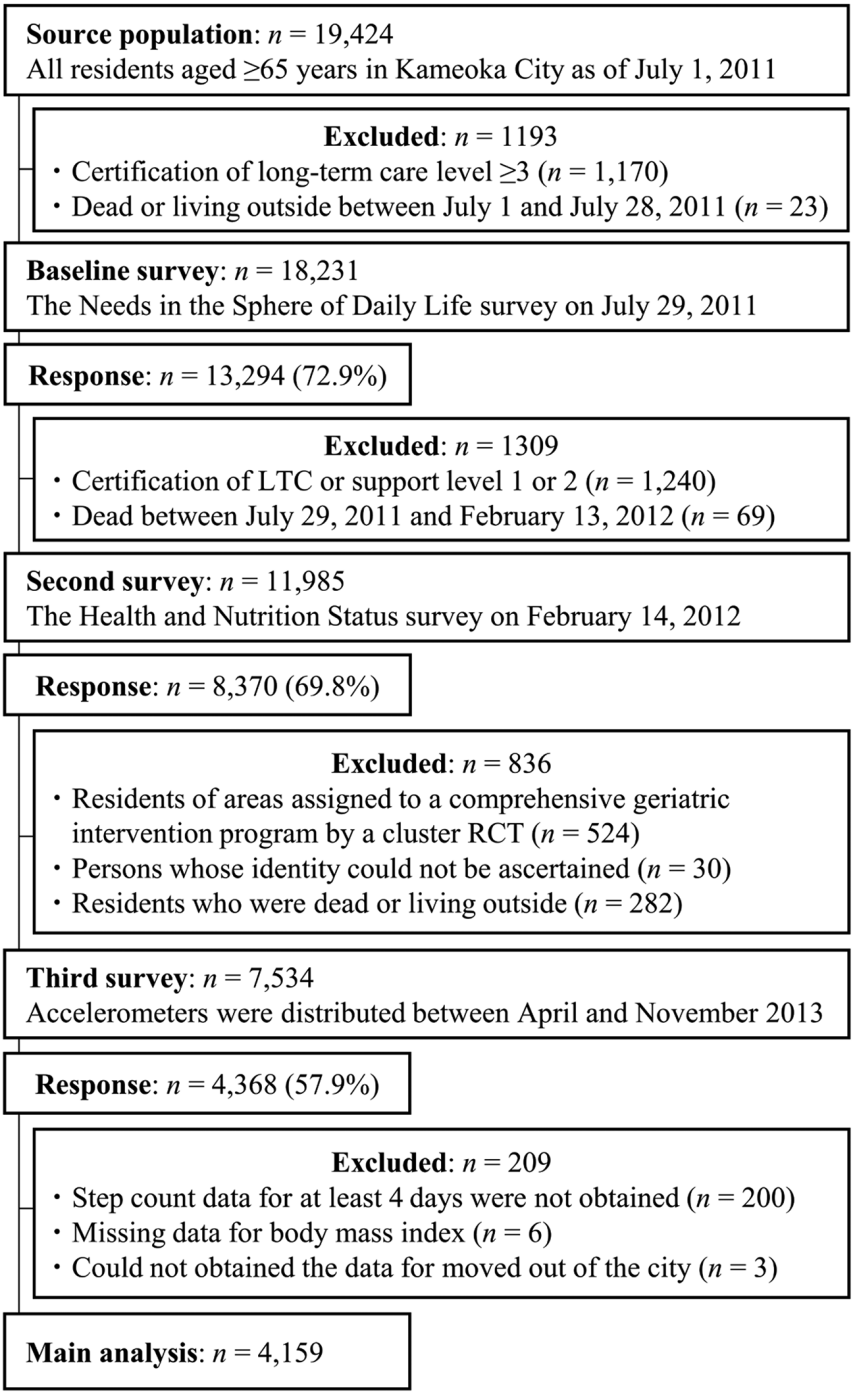
Participant flow diagram for analysis of the relationship between calibrated energy intake and daily step count on all-cause mortality in the Kyoto-Kameoka study. LTC, long-term care; RCT, randomised controlled trial

The Kyoto-Kameoka study was a population-based, prospective cohort study involving individuals aged ≥65 years residing in Kameoka City, Kyoto Prefecture, Japan. Details of this cohort study are explained elsewhere [4, 6, 12–18]. Briefly, the Health and Nutrition Status Survey, which included a food frequency questionnaire (FFQ), was mailed to the residents of Kameoka on 14 February 2012. Of these residents, 8,370 responded to the survey (response rate: 69.8%) and were included as the study participants (Fig. 1). Accelerometers were provided to 7,534 participants between April and November 2013, and 4,368 (rate: 57.9%) obtained a measurement of their daily step counts over at least 1 day. This study was approved by the research ethics committees of the National Institutes of Biomedical Innovation, Health and Nutrition (NIBIOHN-76-2), Kyoto University of Advanced Science (20 − 1), and Kyoto Prefectural University of Medicine (RBMR-E-363). Informed consent forms were received from each participant through mail along with the completed questionnaire. The study results were reported in accordance with Strengthening the Reporting of Observational Studies in Epidemiology-Nutritional Epidemiology guidelines [9].

The exclusion criteria were as follows: (i) participants with no step count data collected using an appropriate accelerometer over a minimum of 4 days, including 1 day holiday (*n* = 200); (ii) participants with missing body mass index (BMI) data, which were required to calculate the calibrated EI (*n* = 6); and (iii) participants who relocated of Kameoka city on an unknown date (*n* = 3). The final analysis included 4,159 participants.

### Energy intake assessment

A previously validated self-administered FFQ comprising 47 food and beverage items was used to estimate the EI from items consumed in the preceding year [4, 17]. The EI calculated from the FFQ was underestimated; thus, the calibrated EI was obtained using a multiple regression model with TEE, determined through the DLW method as the dependent variable, with individual characteristics as the independent variables [4, 6, 17]. The calibration equation was devised to reduce systematic errors in FFQ-based EI estimates and included significant independent variables, such as age, sex, BMI, and FFQ-estimated EI (R^2^ = 0.36) [4, 6, 17]. The validity of the calibrated EI calculated using the equation was verified using repeated measurements of TEE measured through the DLW method in the same group from which the equation was developed (Spearman correlation coefficient = 0.517) [17]. The interclass correlation coefficient on the reproducibility scale for estimates of calibrated EI was 0.921 and 0.945 for women and men, respectively [17].

### Assessment of daily step counts

Step counts were measured using a triaxial accelerometer (EW-NK52; Panasonic Co., Ltd, Osaka, Japan) [12, 13]. Between 1 April and 15 November 2013, the Kameoka residents were mailed an accelerometer with the documents containing the instructions for use. They were requested to wear the device for at least 10 days during waking hours. Each participant was instructed to wear the accelerometer, fastened with a belt, on their waist throughout the day unless sleeping, bathing, or swimming, and resume their usual lifestyle. The daily step count was determined using the manufacturer’s step count algorithm. As the time of wearing the accelerometer could not be determined, the collected data at or below the 1st percentile for step count distribution in people aged 60–69 years based on the results of the National Health and Nutrition Surveys Japan (NHNS-J) (499 and 653 steps for men and women, respectively) were excluded from the analysis as they were categorised as data collected under low compliance [12]. To calculate the daily step count, the total step count measured over a minimum of 4 days, including a 1-day holiday, was obtained [13]. The total step count was divided by the measurement duration (days) to determine the mean daily step count.

### Other covariates

All covariates, including medical history, such as hypertension and diabetes (Table 1), socioeconomic status, smoking status, level of frailty, and educational history, were based on data collected from the questionnaire during the baseline survey. BMI was calculated by dividing self-reported body weight (in kilograms) by height squared (in meters) [14]. Previously, we found no significant difference between BMI calculated from self-reported height and body weight and that objectively measured in a subcohort of the Kyoto-Kameoka Study using clustered random sampling (mean difference: 0.5 kg/m^2^ and 0.4 kg/m^2^ in women and men, respectively) [14]. Pearson’s rank correlation coefficient between BMIs obtained from these measurements and self-reports was 0.912 and 0.916 for women and men, respectively [14]. The interclass correlation coefficients as a reproducibility scale of the self-reported BMI obtained from the baseline and additional surveys were 0.888 and 0.910 for women and men, respectively [14]. Similar results have also been reported in other study in older Japanese adults [19]. The level of frailty was defined as a score of at least 7 of the 25 items on the validated self-administered Kihon Checklist [16]. The ratio of calibrated EI to the predicted basal metabolic rate (calibrated EI/pBMR) was calculated using the pBMR, which was estimated using an equation developed for Japanese adults by Ganpule et al. [20]. We collected information on the following basic characteristics: smoking status (‘Do you smoke?’: almost daily; sometimes; used to but quit; and never); drinking status (‘Do you drink alcohol?’: almost daily, sometimes, almost never, and never); living status (‘What is your family structure?’: living alone, living with family, and others); education attainment (years); socioeconomic status (‘Economically, how does your life feel currently?’: hard, somewhat hard, somewhat easy, and easy); oral status (‘Do you use dentures?’: yes or no); taking medication (number); and chronic disease (‘Do you have a disease (presence of hypertension, stroke, heart disease, diabetes, hyperlipidemia, digestive disease, respiratory disease, urological diseases, and cancer)?’: yes or no). Comorbidity scores were calculated from the data obtained on nine comorbidity statuses. The summed value indicated a total score ranging from 0 (no comorbidity) to 9 (poor status).

**Table 1:** Baseline characteristics of the study participants by calibrated energy intake and daily step count status

	Total (*n* = 4,159)		Calibrated energy intake and daily step count status							
			LEI/LSC (*n* = 1,352)		HEI/LSC (*n* = 1,586)		LEI/HSC (*n* = 471)		HEI/HSC (*n* = 750)	
Age [years] ^a^	72.3	(5.3)	75.5	(5.8)	70.8	(4.2)	72.4	(5.3)	69.3	(3.2)
Women [*n* (%)] ^b^	2024	(48.7)	641	(47.4)	906	(57.1)	151	(32.1)	326	(43.5)
PD ≥ 1,000 people/km^2^ [*n* (%)]^b^	2033	(48.9)	681	(50.4)	800	(50.4)	212	(45.0)	340	(45.3)
Body mass index [kg/m^2^]^a^	22.7	(3.2)	21.4	(2.6)	23.9	(3.5)	21.3	(2.4)	23.1	(2.4)
Current smoker [*n* (%)]^b^	422	(10.1)	146	(10.8)	163	(10.3)	40	(8.5)	73	(9.7)
Alcohol drinker [*n* (%)]^b^	2891	(69.5)	901	(66.6)	1061	(66.9)	344	(73.0)	585	(78.0)
Living alone [*n* (%)] ^b^	480	(11.5)	193	(14.3)	177	(11.2)	49	(10.4)	61	(8.1)
Education ≥ 13 years [*n* (%)]^b^	988	(23.8)	288	(21.3)	370	(23.3)	135	(28.7)	195	(26.0)
HSES [*n* (%)]^b^	1467	(35.3)	495	(36.6)	545	(34.4)	149	(31.6)	278	(37.1)
Denture use [*n* (%)]^b^	2426	(58.3)	882	(65.2)	882	(55.6)	280	(59.4)	382	(50.9)
No medication [*n* (%)]^b^	1020	(24.5)	258	(19.1)	385	(24.3)	125	(26.5)	252	(33.6)
Hypertension [*n* (%)]^b^	1505	(36.2)	489	(36.2)	605	(38.1)	161	(34.2)	250	(33.3)
Stroke [*n* (%)]^b^	123	(3.0)	50	(3.7)	39	(2.5)	12	(2.5)	22	(2.9)
Heart disease [*n* (%)]^b^	461	(11.1)	187	(13.8)	157	(9.9)	50	(10.6)	67	(8.9)
Diabetes [*n* (%)] ^b^	393	(9.4)	141	(10.4)	133	(8.4)	58	(12.3)	61	(8.1)
Hyperlipidemia [*n* (%)]^b^	450	(10.8)	119	(8.8)	223	(14.1)	51	(10.8)	57	(7.6)
Digestive disease [*n* (%)]^b^	353	(8.5)	149	(11.0)	119	(7.5)	41	(8.7)	44	(5.9)
Respiratory disease [*n* (%)]^b^	172	(4.1)	71	(5.3)	64	(4.0)	23	(4.9)	14	(1.9)
Urological diseases [*n* (%)]^b^	274	(6.6)	109	(8.1)	75	(4.7)	45	(9.6)	45	(6.0)
Cancer [*n* (%)]^b^	120	(2.9)	52	(3.8)	48	(3.0)	14	(3.0)	6	(0.8)
No. of chronic diseases^a,c^	0.93	(0.95)	1.01	(1.00)	0.92	(0.94)	0.97	(0.97)	0.75	(0.87)
Frailty [*n* (%)]^b^	1026	(24.7)	471	(34.8)	354	(22.3)	88	(18.7)	113	(15.1)
Calibrated EI [kcal/day]^a^	2172	(281)	2044	(254)	2233	(271)	2131	(245)	2297	(273)
pBMR [kcal/day]^a^	1264	(204)	1214	(199)	1285	(207)	1264	(192)	1311	(195)
Calibrated EI/pBMR^a^	1.73	(0.15)	1.70	(0.15)	1.75	(0.15)	1.70	(0.15)	1.77	(0.14)
Daily step counts [step/day]^a^	4194	(2395)	2805	(1083)	3096	(1054)	7116	(2149)	7187	(2024)
EI/steps [kcal/100 steps/day]^a^	70.0	(42.7)	87.2	(43.6)	83.7	(40.2)	31.9	(7.7)	34.0	(8.5)

EI, energy intake; HEI, high energy intake; HSC, high step counts; HSES, high socioeconomic status; LEI, low energy intake; LSC, low step counts; pBMR, predicted basal metabolic rate; PD, population density. Missing values were supplemented using the multivariate imputation method: family structure (*n* = 266; 6.4%), socioeconomic status (*n* = 170; 4.1%), education attainment (*n* = 400; 9.6%), smoking status (*n* = 150; 3.6%), alcohol drinker (*n* = 124; 3.0%), denture use (*n* = 106; 2.5%), medications (*n* = 285; 6.9%), and frailty status (*n* = 490; 11.8%). Body mass index was calculated as body weight (kg) divided by height squared (m^2^)

^a^Continuous values are shown as mean (standard deviation)

^b^Categorical values are shown as numbers (percentages)

^c^From the data obtained on disease status (including the presence of hypertension, stroke, heart disease, diabetes, hyperlipidemia, digestive disease, respiratory disease, urological diseases, and cancer), the comorbidity scores were summed to obtain a total score ranging from 0 (no comorbidity) to 9 (poor status)

### Mortality status

Participants’ vital status during the follow-up period was assessed using information from the Basic Resident Registration System managed by the Kameoka City Office, covering the period from the date of use of the accelerometer (1 April to 15 November 2013) to 30 November 2016. Data on participants who lost their residency status, moved away from Kameoka, or relocated abroad were deleted from the sample (censoring).

### Statistical analysis

The dose–response relationships between calibrated EI and step count and all-cause mortality were analysed; the calibrated EIs of 2,400–2,600 kcal/day for men and 1,900–2,000 kcal/day for women [6], with daily step counts of 5,000–7,000 [12], had the lowest hazard ratios (HRs). Based on these results, the participants were classified into the following four groups: low EI (LEI)/low step counts (LSC) group (EI: <2,400 kcal/day in men and <1,900 kcal/day in women and steps: <5,000 steps/day), *n* = 1,352; HEI/LSC group (EI: ≥2,400 kcal/day in men and ≥1,900 kcal/day in women and steps: <5,000 steps/day), *n* = 1,586; LEI/HSC group (EI: <2,400 kcal/day in men and <1,900 kcal/day in women and steps: ≥5,000 steps/day), *n* = 471; and HEI/HSC group (EI: ≥2,400 kcal/day in men and ≥1,900 kcal/day in women and steps: ≥5,000 steps/day), *n* = 750.

Continuous variables were expressed as mean and standard deviation, and categorical variables were presented as numbers and percentages. The missing values for covariates were completed using the Multiple Imputation by Chained Equation package in R statistical software, creating five datasets [21]. All missing values were assumed to be missing at random. The participant characteristics in the baseline survey and those included in this study were compared. The restricted cubic spline model was used to evaluate the relationship between calibrated EI and step counts.

The all-cause mortality rate for each group, based on calibrated EI and step count, was presented as the number of events per 1,000 person-years. A multivariable Cox proportional hazards model, which included baseline covariates, was used to adjust for the confounding factors affecting the relationship between EI and step count and mortality risk. The Schoenfeld residuals test was performed to confirm the assumptions of the Cox proportional hazards model, and the results did not reject the null hypothesis (*p*-value = 0.752). Thus, the condition of the proportional hazard was assumed to hold. Multivariable analysis was performed using the following two models. Model 1 was adjusted for age (continuous), sex (female or male), population density (≥ 1,000 or < 1,000 people/km^2^), and the step count assessment season (spring, summer, or autumn). Model 2 was adjusted for BMI (continuous), smoking status (never smoker, past smoker, or current smoker), alcohol consumption (yes or no), living alone (yes or no), educational attainment (< 9, 10–12, or ≥ 13 years), socioeconomic status (high or low), denture use (yes or no), medication use (continuous), number of chronic diseases (continuous), and frailty (yes or no) to Model 1. These adjustment factors were determined from the results of previous studies [6, 12]. The results are presented with the HR and 95% confidence interval (CI), and the HR was calculated based on data from the LEI/LSC group as the standard.

To assess the interaction between outcome and exposure, both additive (relative excess risk due to interaction [RERI]) and multiplicative interactions were calculated using the EI and step count as categorical variables. The following two methods were used to perform sensitivity analysis: (1) mortality events recorded in the first year of follow-up (11 men) were excluded to eliminate the possibility of reverse causality; and (2) the same analysis was conducted using the complete case dataset without missing values.

The relationship between mortality and the combination of calibrated EI and step count was also evaluated using two methods as follows: (i) the calibrated EI per 100 steps/day (EI/100 steps/day) was calculated; (ii) the z-score for both calibrated EI and step count was calculated based on the distribution of the study population, and the calibrated EI z-score and step count z-score were added together to obtain the total z-score. The EI/100 steps and z-scores were classified into quartiles. The linear trend was calculated by considering these exposure variables as continuous variables. To evaluate the relationship between EI/100 steps and z-scores regarding all-cause mortality, we used a restricted spline model based on the distribution of these variables (EI/100 steps: 5th, 25th, 50th, 75th, and 95th percentiles [5 knots]; z-scores: 5th, 50th, and 95th percentiles [3 knots]). These results were expressed as HRs and 95% CIs, and the HR was calculated based on the means of the fourth quartile for EI/100 steps (128 kcal/100 steps) and the first quartile for z-scores (-1.8). Due to the scattering of the data, variables with distributions below 1% or at 99% or higher were excluded from the analysis [12]. The statistical significance of non-linearity was evaluated by comparing the likelihood ratio in the spline model with that in the linear model using the Wald test. A p-value of < 5% was considered to indicate a significant nonlinear relationship between the exposure and outcome.

A two-tailed *p*-value < 0.05 was considered significant for all statistical analyses. All analyses were performed using STATA MP, version 15.0 (StataCorp LP, College Station, Texas, USA) and/or R software 3.4.3 (R Core Team, Vienna, Austria).

## Results

**Fig. 2:**
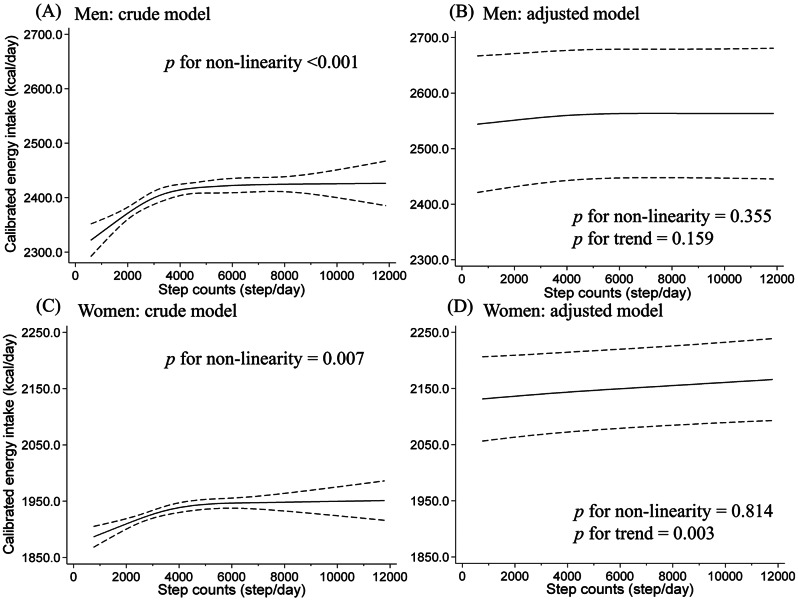
Relationship between calibrated energy intake and daily step counts using a restricted cubic spline model among older adults. (**A**) crude model and (**B**) multivariable adjusted model in 2,107 men and (**C**) crude model and (**D**) multivariable adjusted model in 2,011 women. Because the data were sparse, we truncated the analysis at 12,000 steps/day (99% of the distribution). Solid and dashed lines represent mean calibrated energy intake and 95% confidence intervals, respectively. The adjusted factors were age, population density, season of wear, body mass index, smoking status, alcohol consumption status, family structure, educational attainment, economic status, denture use, medication use, number of chronic diseases, and frailty status

Table 1 shows the participant characteristics in the four groups based on the calibrated EI and step count analysed in the cohort. The mean (standard deviation) calibrated EI and daily step count for the entire population was 2,172 (281) kcal/day and 4,194 (2395) steps/day, respectively. Participants in the HEI/HSC group were younger, with fewer individuals who were frail, lived alone, wore dentures, and had some chronic diseases compared to those in the LEI/LSC group. The HEI/HSC group also included a greater number of individuals who were alcohol drinker and not taking medication. There were fewer mortality events and frail participants included in this study than in the baseline survey (Supplementary Table 1). The unadjusted (crude) model showed a positive dose–response relationship between calibrated EI and steps until approximately 4,000 steps/day (Fig. 2), after which the relationship plateaued (*p* < 0.01 for non-linearity in both sexes with an inverted L-shape relationship). No (men) or slight (women) dose-response relationship was observed after adjusting for confounding factors. Similar trends were observed with uncalibrated EI; however, no significant linear or nonlinear association was observed (Supplementary Fig. 1).

**Fig. 3:**
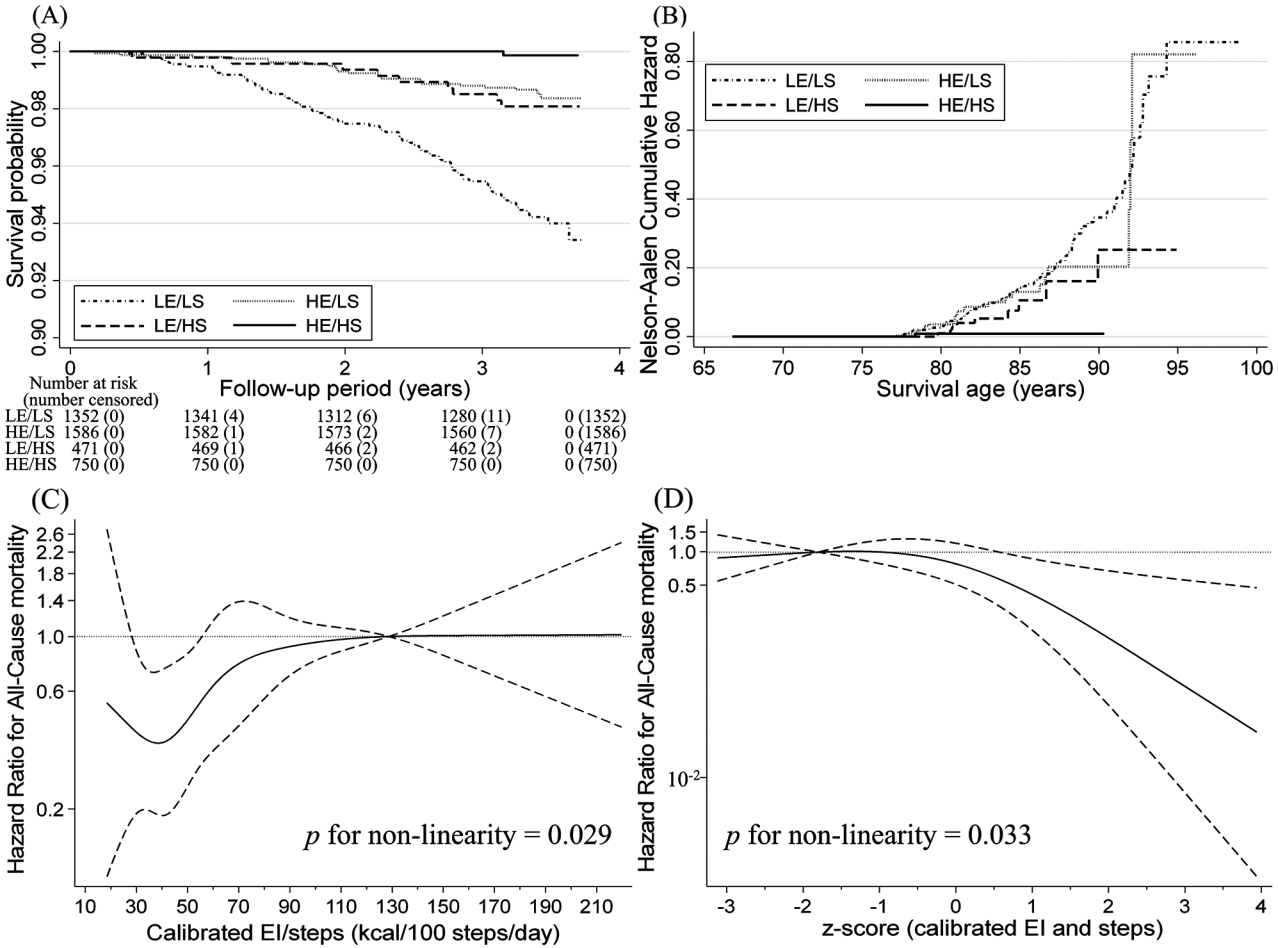
Survival analysis for all-cause mortality according to calibrated energy intake and daily step count status among older adults. (**A**) Multivariable adjusted Kaplan–Meier survival curves using inverse probability weighting and (**B**) Nelson–Aalen cumulative hazard curves using age as the time scale. Solid and dashed lines represent hazard ratios and 95% confidence intervals (CI), respectively, and the hazard ratios based on (**C**) 128 kcal/100 steps for calibrated energy intake (EI) per daily step counts (*n* = 4,071 [*p* for non-linearity = 0.029]) and (**D**) − 1.8 for z-score (*n* = 4,079 [*p* for non-linearity = 0.033]) as the reference (the first or fourth quartile value) were calculated. We estimated that *p* < 0.05 when the 95% CI of the hazard ratio did not exceed 1.00 and *p* ≥ 0.05 when the 95% CI of the hazard ratio exceeded 1.00. The adjusted factors were age, sex, population density, season of wear, body mass index, smoking status, alcohol consumption status, family structure, educational attainment, economic status, denture use, medication use, number of chronic diseases, and frailty status

The relationships between all-cause mortality risk and calibrated EI and step count are shown in Table 2; Fig. 3A and B. The median follow-up period was 3.38 years (interquartile range: 3.28–3.53 years). The total follow-up period was 14,046 person-years, and 111 deaths were reported (2.7%). After adjusting for confounders, the HEI/HSC group had the lowest mortality risk compared to the other groups [LEI/LSC group (reference); HEI/LSC group: HR, 0.71 (95% CI: 0.41–1.23); LEI/HSC group: HR, 0.59 (95% CI: 0.29–1.19); HEI/HSC group: HR, 0.10 (95% CI: 0.01–0.76)]. No significant relationship was found between the mortality risk and calibrated EI and step count interaction. Similar results were obtained in the interaction analysis using continuous variables of step count and EI to enhance interaction test power (*p*-value = 0.101). Sensitivity analysis also produced similar results (Supplementary Tables 2 and 3). The Nelson–Aalen cumulative hazard curves using age as the time scale showed a trend of increasing differences in mortality hazard among the groups based on the calibrated EI and step counts as age increased (Fig. 3B).

**Table 2:** Hazard ratios for calibrated energy intake and daily step count status, and all-cause mortality calculated using the multivariable Cox proportional hazards model

	*n*	Event	PY	Event/1000 PY		Model 1^a^		Model 2^b^	
				Rate	95%CI	HR	95%CI	HR	95%CI
**EI×SC**									
LEI/LSC	1352	78	4500	17.3	(13.9 to 21.6)	1.00	(Ref)	1.00	(Ref)
HEI/LSC	1586	23	5360	4.3	(2.9 to 6.5)	0.72	(0.44 to 1.19)	0.71	(0.41 to 1.23)
LEI/HSC	471	9	1605	5.6	(2.9 to 10.8)	0.54	(0.27 to 1.08)	0.59	(0.29 to 1.19)
HEI/HSC	750	1	2582	0.4	(0.1 to 2.7)	0.10	(0.01 to 0.73)	0.10	(0.01 to 0.76)
**Interaction**									
Additive^c^						-0.16	(-0.90 to 0.58)	-0.20	(-0.89 to 0.49)
* p*-value						0.701		0.753	
Multiplicative^d^				0.28	(0.03 to 2.32)	0.25	(0.03 to 2.12)	0.25	(0.03 to 2.05)
* p*-value				0.238		0.205		0.194	
**EI**									
Low	1823	87	6104	14.3	(11.6 to 17.6)	1.00	(Ref)	1.00	(Ref)
High	2336	24	7942	3.0	(2.0 to 4.5)	0.66	(0.40 to 1.08)	0.64	(0.37 to 1.08)
**SC**									
Low	2938	101	9859	10.2	(8.4 to 12.5)	1.00	(Ref)	1.00	(Ref)
High	1221	10	4187	2.4	(1.3 to 4.4)	0.42	(0.22 to 0.82)	0.45	(0.23 to 0.88)

CI, confidence interval; EI, energy intake; HEI, high energy intake; HR, hazard ratio; HSC, high step counts; LEI, low energy intake; LSC, low step counts; PY, person-years; Ref, reference; RERI, Relative Excess Risk due to Interaction; SC, step counts

^a^Model 1: Adjusted for age, sex, population density, and season of wear

^b^Model 2: Adjusted for Model 1 and body mass index, smoking status, alcohol consumption status, family structure, educational attainment, economic status, denture use, medication use, number of chronic diseases, and frailty status

^c^The additive interaction was calculated as the RERI using the following equation: RERI = (HR [HEI/HSC] − 1) + (HR [HEI/LSC] + HR [LEI/HSC] − 2). The values are shown as RERI (%). It is significant (*p* < 0.05) if the 95% CI of the RERI is not above 0

^d^It is significant (*p* < 0.05) if the 95% CI of the multiplicative interaction is not above 1.00

The relationship between EI/100 steps and z-scores and all-cause mortality is presented in Table 3; Fig. 3C and D. Comparing the second quartile with the fourth quartile of EI/100 steps showed an inverse association with mortality (HR: 0.33, 95% CI: 0.16–0.67). Comparing the fourth quartile with the first quartile of z-scores revealed an inverse association with mortality (HR: 0.25, 95% CI: 0.08–0.75). Analysis stratified based on age and sex showed different age-related associations (Supplementary Tables 4 and 5). The spline model showed that 35–42 kcal/100 steps/day of EI/100 steps was associated with the lowest mortality risk (Fig. 3C). Z-scores of ≥ 0.6 showed a significant inverse association with mortality risk (Fig. 3D).

**Table 3:** Multivariable Cox proportional hazards model for the relationship between calibrated energy intake and daily step count on all-cause mortality

	Q1		Q2		Q3		Q4	
***EI/steps quartile, n***	1037		1041		1038		1043	
Calibrated EI (kcal/day)	2149	(273)	2174	(284)	2166	(284)	2198	(281)
Step counts (steps/day)	7391	(2174)	4497	(764)	3074	(520)	1830	(479)
EI/steps (kcal/100 steps)	30.7	(6.5)	48.9	(5.2)	71.3	(7.9)	128.8	(41.2)
z-score	1.28	(1.41)	0.21	(1.14)	-0.46	(1.11)	-1.03	(1.18)
No of deaths/PY	11	(3553)	9	(3538)	31	(3501)	60	(3454)
Rate/1000 PY (95% CI)	3.1	(1.7 to 5.6)	2.5	(1.3 to 4.9)	8.9	(6.2 to 12.6)	17.4	(13.5 to 22.4)
Model 1^a^	0.47	(0.24 to 0.91)	0.32	(0.16 to 0.66)	0.83	(0.53 to 1.29)	1.00	(Ref)
Model 2^b^	0.51	(0.26 to 1.01)	0.33	(0.16 to 0.67)	0.82	(0.52 to 1.29)	1.00	(Ref)
***z-score quartile, n***	1043		1038		1040		1038	
Calibrated EI (kcal/day)	1999	(245)	2140	(247)	2219	(244)	2330	(277)
Step counts (steps/day)	2434	(1081)	3265	(1301)	4291	(1504)	6796	(2664)
EI/steps (kcal/100 steps)	99.8	(48.6)	78.6	(39.8)	60.3	(30.1)	41.2	(23.3)
z-score	-1.80	(0.63)	-0.51	(0.26)	0.38	(0.27)	1.94	(0.96)
No of deaths (PY)	68	(3447)	27	(3518)	12	(3527)	4	(3553)
Rate/1000 PY (95% CI)	19.7	(15.6 to 25.0)	7.7	(5.3 to 11.2)	3.4	(1.9 to 6.0)	1.1	(0.4 to 3.0)
Model 1^a^	1.00	(Ref)	0.85	(0.53 to 1.35)	0.57	(0.30 to 1.10)	0.25	(0.09 to 0.74)
Model 2^b^	1.00	(Ref)	0.85	(0.53 to 1.39)	0.59	(0.30 to 1.17)	0.25	(0.08 to 0.75)

CI, confidence interval; EI, energy intake; HEI, high energy intake; HR, hazard ratio; HSC, high step counts; LEI, low energy intake; LSC, low step counts; PY, person-years; Q, quantile; Ref, reference; RERI, Relative Excess Risk due to Interaction; SC, step counts. Q1 through Q4 include calibrated energy intake per 100 step counts/day for calibrated energy intake and step counts of < 40.4, 40.4–58.5, 58.6–86.3, and ≥ 86.4 kcal/100 steps, respectively. Q1 through Q4 include z-score for calibrated energy intake and step counts of <-0.99, -0.99–-0.07, -0.06–0.88, and ≥ 0.89 scores, respectively

^a^Model 1: Adjusted for age, sex, population density, and season of wear

^b^Model 2: Adjusted for Model 1 and body mass index, smoking status, alcohol consumption status, family structure, educational attainment, economic status, denture use, medication use, number of chronic diseases, and frailty status

## Discussion

To the best of our knowledge, this is the first study to examine the relationship between calibrated EI and step count and mortality risk. The results showed that the lowest relationship with all-cause mortality was in the HEI/HSC group. However, no interaction was found between mortality risk, calibrated EI, and step count. The mortality risk HR was lowest at 35–42 kcal/100 steps/day, suggesting that extremely high (≥56 kcal/100 steps/day) or low (<28 kcal/100 steps/day) EI/100 steps are not inversely associated with mortality. Thus, our findings may be useful in determining the optimal EI according to the recommended physical activity, including those from the World Health Organisation (WHO), the United States, and Japan [18] in older individuals whose TEE begins to decline (changing energy balance) [11].

In the 1950s, Mayer et al. reported a U-shape relationship between physical activity and EI in middle-aged people and hypothesised that a physical activity threshold exists that can regulate EI [22]. Subsequent longitudinal studies have also shown a J-shape relationship between physical activity and EI, with the group with the lowest physical activity having higher EI and body weight [23]. Our study demonstrated that older adults with approximately <4,000 steps per day had lower EI regardless of sex, although these relationships disappeared after adjusting for confounders. The older adults with the lowest physical activity assessed using the DLW method had lower TEE and BMI than those in the highest third [24], suggesting that those with low physical activity also had low EI. A 7-year comparison of baseline estimates of energy expenditure and fat-free mass from DLW in American older adults showed a decrease in men but no change in women [25]. Since habitual EI can be estimated from the body composition [23], the decrease in energy expenditure from physical activity in older adults may be related to a decrease in EI. A meta-analysis of 98 studies, including 4,972 individuals with DLW methods, showed that more economically developed countries had higher TEE than less economically developed ones [26]. However, no significant difference was found in TEE adjusted for body weight and age [26]. These studies support our results, and suggest that improving physical activity is important in enabling older adults to obtain necessary nutrients despite a loss of appetite.

Even among individuals with equivalent TEE, there are those with a higher basal metabolism but less physical activity (obese individuals) and those with a lower basal metabolism but more physical activity (active individuals) [27]. This increases the importance of evaluating the long-term outcome relationship considering both physical activity and EI. Our findings show that individuals with high calibrated EI and step counts had the most inverse association with mortality compared to the other groups. Epidemiological studies investigating the relationship between the combination of diet quality and physical activity and mortality risk found that a good quality diet and high levels of physical activity were strongly inversely associated with mortality risk. However, mortality risk was not associated with the interaction between diet quality and physical activity [28]. These findings are similar to those in this study. The daily step count dose–response curve at which the HR for mortality plateaued among older adults was approximately 6,000–8,000 and 5,000–7,000 steps in a previous study from 15 international cohorts [29] and our cohort study [12], respectively. The optimal step counts associated with the lowest mortality risk show a similarity between these studies [12, 29] and meet the target value outlined in physical activity guidelines for older adults by the WHO, the United States, and Japan [18]. According to our study findings, the optimal EI for older individuals with 6,000 steps/day would be 2,100–2,520 kcal/day. This is similar to our previously reported optimal EI with the lowest risk of death [6] and the average TEE values in older adults aged ≥65 years reported by the International DLW database group [11]. A U-shaped relationship between BMI, which is a long-term indicator of energy balance, and death risk has been reported [3]. This suggests that deviations from optimal body mass resulting from positive or inverse fluctuations in energy balance have an inverse impact on outcomes. We previously reported that biomarker-calibrated EI had an inverse association with death, although this relationship was not observed when adjusting for BMI [6]. Interestingly, the results of our analysis remained significant even after adjusting for BMI in this study. This suggests that the inverse relationship between EI and physical activity and death is influenced by factors other than energy balance, and evaluating both of these factors may explain their benefit regarding health outcomes that cannot be assessed using BMI alone.

Understanding of the mechanisms underlying the inverse associations between EI and step count and mortality risk, independent of BMI, is limited. However, two potential mechanisms should be considered based on the results of several previous studies. First, physical activity has beneficial effects on metabolism. In previous studies involving individuals with excessive EI and reduced physical activity due to restrictions on step counts, insulin sensitivity disorders developed before body composition changes occurred [30]. Second, HEI and high energy expenditure, indicative of high energy flux, have beneficial effects. A study using a metabolism chamber found that high energy flux showed the greatest relative increase in fat oxidation instead of protein oxidation compared with other approaches, including energy restriction and low physical activity [31]. In the case of older adults who habitually perform aerobic exercise and have long-term high energy flux, reducing exercise and EI diminished the energy flux, leading to decreased β-adrenalin–receptor stimulation, resting metabolic rate, and skeletal sympathetic nervous system activity [32]. High energy flux improves the individual’s metabolic profile, including levels of glucose and fats, with no changes in body weight [33]. The above points support our finding that the combination of EI and physical activity is inversely associated with mortality risk, even when adjusting for BMI. However, these studies had a short-term duration and only evaluated the rapid metabolic responses; therefore, further long-term studies are required to understand the cause-effect and temporal relationships.

Although the HEI/HSC group had the lowest all-cause mortality compared to other groups, no significant interaction was observed between the calibrated EI and step count with mortality. The biological mechanism underlying this lack of association is not well understood. Diet and physical activity are believed to be interdependent regarding biological processes, as adjusting for physical activity in statistical models changes the relationship between diet and health outcomes [34]. However, independent of biological mechanisms, reasons that we could not observe an interaction between EI and physical activity may also be influenced by methodological reasons, such as the small number of mortality events due to the comparatively short observation period and the small sample size. Cut-off points for the calibrated EI and step count dichotomies were chosen based on previous mortality data reports for this cohort. The four calibrated EI/step count categories exhibit highly variable numbers of deaths (e.g., only one death in the HEI/HSC category). In this case, the HR interaction test power will likely be relatively limited. Although similar results were obtained in the interaction analysis using step count and EI continuous variables to enhance the interaction test power, well-designed further prospective studies with longer observation periods and more accurately assessed EI and physical activity are required to re-evaluate our results.

A strength of this study lies in the large-scale cohort design involving older people living in the community, where step counts and EI were evaluated using an accelerometer and DLW-calibration approach, respectively, minimising bias due to the self-reporting of exposure variables. Nevertheless, this study had some methodological limitations. First, the EI calibration formula was prepared based on the TEE of a subsample of participants with stable body weights in the Kyoto-Kameoka Study [4, 17]. Therefore, if participants with unstable body weights were included in the analysed population, there may be systematic errors in the estimate of calibrated EI. Second, step count data could not be collected from all participants provided with accelerometers. Differences were observed in the characteristics and mortality risk between the participants in this study and those in the baseline survey. This suggests that participants in this study were more health-conscious than older adults in the general population, thereby resulting in a potential selection bias. Additionally, caution may be needed when generalising our main findings to other populations. For instance, the average BMI in our cohort was 22.7 kg/m^2^, which is significantly lower than that in the United States and European cohorts that have been used for similar purposes [35]. Particularly, HEI could be beneficial in the studied population; however, it may be detrimental in populations with high rates of obesity or overweight [7, 36]. Third, data about accelerometer wearing time could not be obtained; hence, the inclusion of days when the accelerometer compliance rate was low may have led to the underestimation of step counts. Nonetheless, the mean daily step counts estimated in this study did not differ markedly from those in the NHNS-J [12]. Fourth, data on mortality factors were unavailable; thus, the different causes of death that were inversely associated with the combination of EI and step count could not be determined. Furthermore, the observation period was relatively short. The HR estimated from the analysis changes with time; considering that the results were only estimated within a shorter observation period, the potential exists for overestimation of the relationships between exposure factors and outcomes and inversion of the cause-effect relationship [37]. However, the proportional hazards for the relationships between exposure and mortality risk were confirmed. Similar results were observed in a sensitivity analysis that excluded the mortality events occurring after 1 year of follow-up. Finally, despite adjusting for confounding factors, the occurrence of residual confounding in the relationships of calibrated EI and step counts with all-cause mortality still exists. Because these factors can be confounders, body composition, such as body fat and skeletal muscle mass should be considered when considering energy balance and food insecurity and access to food should be considered when examining the relationship between physical activity and EI.

Recently, objective methods, including triaxial accelerometers, have been used to assess habitual physical activity; however, self-reported dietary assessment methods, such as FFQ and dietary records, are still used to assess energy and nutrient intake. In prospective cohort studies using EI estimated from self-reported dietary assessment methods, no consistent results have been reported regarding the association between EI and the risk of death in older adults [38, 39]. To verify the combined effect of EI and physical activity on mortality risk, it is necessary to accurately assess exposure variables, particularly EI, because we have previously reported that DLW-calibrated EI [6] and self-reported physical activity [18], as opposed to uncalibrated EI [6], were associated with mortality. Therefore, to evaluate the interaction between diet and physical activity, it is crucial to evaluate these exposure variables using methods that reduce systematic error as much as possible, such as accelerometer or biomarker-calibration approach. This could partially resolve the systematic errors that have hindered epidemiological study for several decades and may prove useful in bridging the knowledge gap in diet–physical activity interaction on human health and lifespan.

## Conclusion

The combination of EI and step counts showed a strong inverse association with mortality risk in older adults. We showed that the HR for mortality risk was the lowest at 35–42 kcal/100 steps/day, suggesting that extremely high (≥56 kcal/100 steps/day) or low (<28 kcal/100 steps/day) EI/100 steps are not inversely associated with mortality. Therefore, both EI and physical activity should be evaluated to potentially prolong the lifespan of older adults.

### Electronic supplementary material

Below is the link to the electronic supplementary material.

**Supplementary Material 1:** Strengthening the Reporting of Observational Studies in Epidemiology - nutritional epidemiology (STROBE-nut) checklist

**Supplementary Material 2:** **Supplementary Table 1.** Baseline characteristics of residents and additional survey results in the Kyoto-Kameoka Study. **Supplementary Table 2.** Results of sensitivity analysis for the relationship between calibrated energy intake and daily step count on all-cause mortality after excluding participants with an event in the first year of follow-up. **Supplementary Table 3.** Results of sensitivity analysis for the relationship between calibrated energy intake and daily step count on all-cause mortality using complete case data. **Supplementary Table 4.** Hazard ratios for calibrated energy intake per 100 step counts and all-cause mortality calculated using age- and sex-stratified multivariable Cox proportional hazards analysis. **Supplementary Table 5.** Hazard ratios for z-score for calibrated energy intake and step counts and all-cause mortality calculated using ageand sex-stratified multivariable Cox proportional hazards analysis. **Supplemental Figure 1.** Relationship between uncalibrated energy intake and daily step counts using a restricted cubic spline model among older adults

## Data Availability

All data sharing and collaboration requests should be directed to the corresponding author (d2watanabe@nibiohn.go.jp), TY (t-yoshida@nibiohn.go.jp), and YY (yamaday@nibiohn.go.jp).

## List of abbreviations

BMI: body mass index
CI: confidence interval
DLW: doubly labelled water
EI: energy intake
FFQ: food frequency questionaries
HEI: high EI
HR: Hazard Ratio
HSC: high step counts
LEI: low EI
LSC: low step counts
NHNS-J: National Health and Nutrition Surveys Japan
pBMR: predicted basal metabolic rate
RERI: relative excess risk due to interaction
TEE: total energy expenditure

## References

[1] GBD 2019 Risk Factors Collaborators (2020). Global burden of 87 risk factors in 204 countries and territories, 1990–2019: a systematic analysis for the global burden of Disease Study 2019. Lancet.

[2] Hall KD, Sacks G, Chandramohan D, Chow CC, Wang YC, Gortmaker SL, Swinburn BA (2011). Quantification of the effect of energy imbalance on bodyweight. Lancet.

[3] Global BMIM, Di Collaboration E, Bhupathiraju Sh N, Wormser D, Gao P, Kaptoge S, Berrington de Gonzalez A, Cairns BJ, Huxley R, Jackson Ch L (2016). Body-mass index and all-cause mortality: individual-participant-data meta-analysis of 239 prospective studies in four continents. Lancet.

[4] Watanabe D, Nanri H, Sagayama H, Yoshida T, Itoi A, Yamaguchi M, Yokoyama K, Watanabe Y, Goto C, Ebine N (2019). Estimation of Energy Intake by a food frequency questionnaire: calibration and validation with the doubly labeled Water Method in Japanese Older people. Nutrients.

[5] Neuhouser ML, Tinker L, Shaw PA, Schoeller D, Bingham SA, Horn LV, Beresford SA, Caan B, Thomson C, Satterfield S (2008). Use of recovery biomarkers to calibrate nutrient consumption self-reports in the women’s Health Initiative. Am J Epidemiol.

[6] Watanabe D, Yoshida T, Watanabe Y, Kimura M, Yamada Y, Kyoto-Kameoka Study G (2022). Doubly labelled water-calibrated energy intake associations with mortality risk among older adults. J Cachexia Sarcopenia Muscle.

[7] Zheng C, Beresford SA, Van Horn L, Tinker LF, Thomson CA, Neuhouser ML, Di C, Manson JE, Mossavar-Rahmani Y, Seguin R (2014). Simultaneous association of total energy consumption and activity-related energy expenditure with risks of Cardiovascular Disease, cancer, and Diabetes among postmenopausal women. Am J Epidemiol.

[8] Prentice RL, Howard BV, Van Horn L, Neuhouser ML, Anderson GL, Tinker LF, Lampe JW, Raftery D, Pettinger M, Aragaki AK (2021). Nutritional epidemiology and the women’s Health Initiative: a review. Am J Clin Nutr.

[9] Lachat C, Hawwash D, Ocke MC, Berg C, Forsum E, Hornell A, Larsson C, Sonestedt E, Wirfalt E, Akesson A (2016). Strengthening the reporting of Observational studies in Epidemiology-Nutritional Epidemiology (STROBE-nut): an extension of the STROBE Statement. PLoS Med.

[10] Celis-Morales CA, Perez-Bravo F, Ibanez L, Salas C, Bailey ME, Gill JM (2012). Objective vs. self-reported physical activity and sedentary time: effects of measurement method on relationships with risk biomarkers. PLoS ONE.

[11] Pontzer H, Yamada Y, Sagayama H, Ainslie PN, Andersen LF, Anderson LJ, Arab L, Baddou I, Bedu-Addo K, Blaak EE (2021). Daily energy expenditure through the human life course. Science.

[12] Watanabe D, Yoshida T, Watanabe Y, Yamada Y, Miyachi M, Kimura M (2023). Dose-response relationships between objectively measured Daily steps and Mortality among Frail and Non-frail older adults. Med Sci Sports Exerc.

[13] Watanabe D, Yoshida T, Watanabe Y, Yamada Y, Kimura M, Group KS (2020). Objectively measured Daily Step counts and Prevalence of Frailty in 3,616 older adults. J Am Geriatr Soc.

[14] Watanabe D, Yoshida T, Watanabe Y, Yamada Y, Kimura M, Kyoto-Kameoka Study G (2020). A U-Shaped relationship between the prevalence of Frailty and Body Mass Index in Community-Dwelling Japanese older adults: the Kyoto-Kameoka Study. J Clin Med.

[15] Yamada Y, Nanri H, Watanabe Y, Yoshida T, Yokoyama K, Itoi A, Date H, Yamaguchi M, Miyake M, Yamagata E (2017). Prevalence of Frailty assessed by Fried and Kihon Checklist Indexes in a prospective cohort study: design and demographics of the Kyoto-Kameoka Longitudinal Study. J Am Med Dir Assoc.

[16] Watanabe D, Yoshida T, Watanabe Y, Yamada Y, Miyachi M, Kimura M (2022). Validation of the Kihon Checklist and the frailty screening index for frailty defined by the phenotype model in older Japanese adults. BMC Geriatr.

[17] Watanabe D, Yoshida T, Yoshimura E, Nanri H, Goto C, Ishikawa-Takata K, Ebine N, Fujita H, Kimura M, Yamada Y et al. Doubly labelled water-calibration approach attenuates the underestimation of energy intake calculated from self-reported dietary assessment data in Japanese older adults. Public Health Nutr 2021:1–11. 10.1017/S1368980021003785 PMC9991602 34472428

[18] Watanabe D, Yamada Y, Yoshida T, Watanabe Y, Hatamoto Y, Fujita H, Miyachi M, Kimura M (2022). Association of the interaction between physical activity and sitting time with mortality in older Japanese adults. Scand J Med Sci Sports.

[19] Yazawa A, Inoue Y, Kondo N, Miyaguni Y, Ojima T, Kondo K, Kawachi I (2020). Accuracy of self-reported weight, height and body mass index among older people in Japan. Geriatr Gerontol Int.

[20] Ganpule AA, Tanaka S, Ishikawa-Takata K, Tabata I (2007). Interindividual variability in sleeping metabolic rate in Japanese subjects. Eur J Clin Nutr.

[21] Buuren SV, Groothuis-Oudshoorn K (2011). Mice: multivariate imputation by chained equations in R. J Stat Softw.

[22] Mayer J, Roy P, Mitra KP (1956). Relation between caloric intake, body weight, and physical work: studies in an industrial male population in West Bengal. Am J Clin Nutr.

[23] Shook RP, Hand GA, Drenowatz C, Hebert JR, Paluch AE, Blundell JE, Hill JO, Katzmarzyk PT, Church TS, Blair SN (2015). Low levels of physical activity are associated with dysregulation of energy intake and fat mass gain over 1 year. Am J Clin Nutr.

[24] Manini TM, Everhart JE, Patel KV, Schoeller DA, Colbert LH, Visser M, Tylavsky F, Bauer DC, Goodpaster BH, Harris TB (2006). Daily activity energy expenditure and mortality among older adults. JAMA.

[25] Cooper JA, Manini TM, Paton CM, Yamada Y, Everhart JE, Cummings S, Mackey DC, Newman AB, Glynn NW, Tylavsky F (2013). Longitudinal change in energy expenditure and effects on energy requirements of the elderly. Nutr J.

[26] Dugas LR, Harders R, Merrill S, Ebersole K, Shoham DA, Rush EC, Assah FK, Forrester T, Durazo-Arvizu RA, Luke A (2011). Energy expenditure in adults living in developing compared with industrialized countries: a meta-analysis of doubly labeled water studies. Am J Clin Nutr.

[27] Melanson EL (2017). The effect of exercise on non-exercise physical activity and sedentary behavior in adults. Obes Rev.

[28] Ding D, Van Buskirk J, Nguyen B, Stamatakis E, Elbarbary M, Veronese N, Clare PJ, Lee IM, Ekelund U, Fontana L. Physical activity, diet quality and all-cause Cardiovascular Disease and cancer mortality: a prospective study of 346 627 UK Biobank participants. Br J Sports Med 2022. 10.1136/bjsports-2021-105195 35811091

[29] Paluch AE, Bajpai S, Bassett DR, Carnethon MR, Ekelund U, Evenson KR, Galuska DA, Jefferis BJ, Kraus WE, Lee IM (2022). Daily steps and all-cause mortality: a meta-analysis of 15 international cohorts. Lancet Public Health.

[30] Knudsen SH, Hansen LS, Pedersen M, Dejgaard T, Hansen J, Hall GV, Thomsen C, Solomon TP, Pedersen BK, Krogh-Madsen R (2012). Changes in insulin sensitivity precede changes in body composition during 14 days of step reduction combined with overfeeding in healthy young men. J Appl Physiol (1985).

[31] Nas A, Busing F, Hagele FA, Hasler M, Muller MJ, Bosy-Westphal A (2020). Impact of energy turnover on fat balance in healthy young men during energy balance, energetic restriction and overfeeding. Br J Nutr.

[32] Bell C, Day DS, Jones PP, Christou DD, Petitt DS, Osterberg K, Melby CL, Seals DR (2004). High energy flux mediates the tonically augmented beta-adrenergic support of resting metabolic rate in habitually exercising older adults. J Clin Endocrinol Metab.

[33] Gregory AH, Steven NB (2014). Energy Flux and its role in obesity and metabolic Disease. Eur Endocrinol.

[34] Baranowski T (2004). Why combine diet and physical activity in the same international research society?. Int J Behav Nutr Phys Act.

[35] NCD Risk Factor Collaboration (2019). Rising rural body-mass index is the main driver of the global obesity epidemic in adults. Nature.

[36] Prentice RL, Aragaki AK, Manson JE, Schoeller DA, Tinker LF, Mossavar-Rahmani Y, Wallace RB, LaMonte MJ, Tooze JA, Johnson KC (2023). Total energy expenditure as assessed by doubly labeled water and all-cause mortality in a cohort of postmenopausal women. Am J Clin Nutr.

[37] Matthews CE, Troiano RP, Salerno EA, Berrigan D, Patel SB, Shiroma EJ, Saint-Maurice PF (2020). Exploration of confounding due to Poor Health in an accelerometer-mortality study. Med Sci Sports Exerc.

[38] Lee PH, Chan CW (2016). Energy intake, energy required and mortality in an older population. Public Health Nutr.

[39] Willcox BJ, Yano K, Chen R, Willcox DC, Rodriguez BL, Masaki KH, Donlon T, Tanaka B, Curb JD (2004). How much should we eat? The association between energy intake and mortality in a 36-year follow-up study of Japanese-American men. J Gerontol A Biol Sci Med Sci.

