# Supplementary material for: Association between doubly labelled water-calibrated energy intake and objectively measured physical activity with mortality risk in older adults
Source: Int J Behav Nutr Phys Act. 2023 Dec 25;20:150. doi: 10.1186/s12966-023-01550-x (PMC10749503; doi:10.1186/s12966-023-01550-x)
Supplement: Supplementary file 2 — Supplementary Material 2: Supplementary Table 1. Baseline characteristics of residents and additional survey results in the Kyoto-Kameoka Study. Supplementary Table 2. Results of sensitivity analysis for the relationship between calibrated energy intake and daily step count on all-cause mortality after excluding participants with an event in the first year of follow-up. Supplementary Table 3. Results of sensitivity analysis for the relationship between calibrated energy intake and daily step count on all-cause mortality using complete case data. Supplementary Table 4. Hazard ratios for calibrated energy intake per 100 step counts and all-cause mortality calculated using age- and sex-stratified multivariable Cox proportional hazards analysis. Supplementary Table 5. Hazard ratios for z-score for calibrated energy intake and step counts and all-cause mortality calculated using ageand sex-stratified multivariable Cox proportional hazards analysis. Supplemental Figure 1. Relationship between uncalibrated energy intake and daily step counts using a restricted cubic spline model among older adults [file 12966_2023_1550_MOESM2_ESM.docx]

Supporting information

“Association between doubly labelled water-calibrated energy intake and objectively measured physical activity with mortality risk in older adults”

Authors: Daiki Watanabe, Tsukasa Yoshida, Yuya Watanabe, Yosuke Yamada, Motohiko Miyachi, and Misaka Kimura

**SUPPLEMENTARY TABLES**

**Supplementary Table 1.** Baseline characteristics of residents and additional survey results in the Kyoto-Kameoka Study

**Supplementary Table 2.** Results of sensitivity analysis for the relationship between calibrated energy intake and daily step count on all-cause mortality after excluding participants with an event in the first year of follow-up

**Supplementary Table 3.** Results of sensitivity analysis for the relationship between calibrated energy intake and daily step count on all-cause mortality using complete case data

**Supplementary Table 4.** Hazard ratios for calibrated energy intake per 100 step counts and all-cause mortality calculated using age- and sex-stratified multivariable Cox proportional hazards analysis

**Supplementary Table 5.** Hazard ratios for z-score for calibrated energy intake and step counts and all-cause mortality calculated using age- and sex-stratified multivariable Cox proportional hazards analysis

**SUPPLEMENTAL FIGURES**

**Supplemental Figure 1.** Relationship between uncalibrated energy intake and daily step counts using a restricted cubic spline model among older adults

**Supplementary Table 1**. Characteristics of participants with baseline and additional surveys and accelerometer study in the Kyoto-Kameoka Study

|  | Only first survey | | | |  | First and second survey | |  | Accelerometer study | |
| --- | --- | --- | --- | --- | --- | --- | --- | --- | --- | --- |
|  | All participants (*n* = 13,294) | | Participants without LTC (*n* = 12,054) | |  | All participants (*n* = 8,319) | |  | Included participants (*n* = 4,159) | |
| Age [years] ^a^ | 74.5 | (6.9) | 73.7 | (6.3) |  | 73.6 | (6.2) |  | 72.3 | (5.3) |
| Women [*n* (%)] ^b^ | 7337 | (55.2) | 6456 | (53.6) |  | 4412 | (53.0) |  | 2024 | (48.7) |
| PD ≥1,000 people/km^2^ [*n* (%)] ^b^ | 5917 | (44.5) | 5377 | (44.6) |  | 3814 | (45.8) |  | 2033 | (48.9) |
| Body mass index [kg/m^2^] ^a^ | 22.5 | (3.6) | 22.6 | (3.5) |  | 22.6 | (3.5) |  | 22.7 | (3.2) |
| Living alone [*n* (%)] ^b^ | 1695 | (12.8) | 1427 | (11.8) |  | 966 | (11.6) |  | 480 | (11.5) |
| HSES [*n* (%)] ^b^ | 4228 | (31.8) | 3832 | (31.8) |  | 2770 | (33.3) |  | 1467 | (35.3) |
| Education ≥13 years [*n* (%)] ^b^ | 2567 | (19.3) | 2387 | (19.8) |  | 1745 | (21.0) |  | 988 | (23.8) |
| Current smoker [*n* (%)] ^b^ | 1397 | (10.5) | 1346 | (11.2) |  | 867 | (10.4) |  | 422 | (10.1) |
| Alcohol drinker [*n* (%)] ^b^ | 8277 | (62.3) | 7776 | (64.5) |  | 5470 | (65.8) |  | 2891 | (69.5) |
| Denture use [*n* (%)] ^b^ | 8418 | (63.3) | 7464 | (61.9) |  | 5127 | (61.6) |  | 2426 | (58.3) |
| No medication [*n* (%)] ^b^ | 2604 | (19.6) | 2516 | (20.9) |  | 1826 | (21.9) |  | 1020 | (24.5) |
| No. of chronic diseases ^a,c^ | 0.96 | (1.00) | 0.93 | (0.97) |  | 0.95 | (0.97) |  | 0.93 | (0.95) |
| Frailty [*n* (%)] ^b^ | 5321 | (44.4) | 4229 | (39.2) |  | 2768 | (36.9) |  | 1026 | (24.7) |
| Mortality [event/1000 PY] ^d^ | 27.2 | | 21.4 | |  | 17.1 | |  | 7.9 | |
| [95% CI] ^d^ | (26.0 to 28.5) | | (20.3 to 22.6) | |  | (15.9 to 18.4) | |  | (6.6 to 9.5) | |

LTC, long-term care; PD, population density; HSES, high socioeconomic status; PY, person-years; CI, confidence interval

Missing values were supplemented using the multivariate imputation method in all participants (*n* = 13,294): body mass index (*n* = 1,039; 7.8%), family structure (*n* = 1,119; 8.4%), socioeconomic status (*n* = 730; 5.5%), education attainment (*n* = 1,895; 14.3%), smoking status (*n* = 702; 5.3%), alcohol drinker (*n* = 604; 4.5%), denture use (*n* = 405; 3.0%), medications (*n* = 1,140; 8.6%), and frailty status (*n* = 1,722; 13.0%). Body mass index was calculated as body weight (kg) divided by height squared (m^2^).

^a^ Continuous values are shown as mean (standard deviation).

^b^ Categorical values are shown as number (percentage).

^c^ From the data obtained on disease status (including the presence of hypertension, stroke, heart disease, diabetes, hyperlipidemia, digestive disease, respiratory disease, urological diseases, and cancer), the comorbidity scores were summed to obtain a total score ranging from 0 (no comorbidity) to 9 (poor status).

^d^ Mortality was calculated using data from July 30, 2011, to November 30, 2016. Mortality risk is shown as rate (95% CI) per 1000 person-years.

**Supplementary Table 2.** Results of sensitivity analysis for the relationship between calibrated energy intake and daily step count on all-cause mortality after excluding participants with an event in the first year of follow-up

|  | *n* | Event | PY | Event/1000 PY | | Model 1^a^ | | Model 2^b^ | |
| --- | --- | --- | --- | --- | --- | --- | --- | --- | --- |
|  |  |  |  | Rate | 95%CI | HR | 95%CI | HR | 95%CI |
| **EI×SC** |  | | | | | | | | |
| LEI/LSC | 1345 | 71 | 4495 | 15.8 | (12.5 to 19.9) | 1.00 | (Ref) | 1.00 | (Ref) |
| HEI/LSC | 1583 | 20 | 5358 | 3.7 | (2.4 to 5.8) | 0.68 | (0.40 to 1.16) | 0.67 | (0.38 to 1.20) |
| LEI/HSC | 470 | 8 | 1604 | 5.0 | (2.5 to 10.0) | 0.53 | (0.25 to 1.12) | 0.58 | (0.27 to 1.23) |
| HEI/HSC | 750 | 1 | 2582 | 0.4 | (0.1 to 2.7) | 0.11 | (0.01 to 0.80) | 0.11 | (0.01 to 0.82) |
| *Interaction* |  |  |  |  |  |  |  |  |  |
| Additive^c^ |  |  |  |  |  | -0.10 | (-0.85 to 0.64) | -0.14 | (-0.83 to 0.55) |
| *p*-value |  |  |  |  | | 0.791 | | 0.843 | |
| Multiplicative^d^ |  |  |  | 0.33 | (0.04 to 2.79) | 0.30 | (0.04 to 2.53) | 0.28 | (0.03 to 2.41) |
| *p*-value |  |  |  | 0.309 | | 0.267 | | 0.249 | |
| **EI** |  |  |  |  |  |  |  |  |  |
| Low | 1815 | 79 | 6099 | 13.0 | (10.4 to 16.1) | 1.00 | (Ref) | 1.00 | (Ref) |
| High | 2333 | 21 | 7940 | 2.6 | (1.7 to 4.1) | 0.63 | (0.37 to 1.06) | 0.60 | (0.34 to 1.06) |
| **SC** |  |  |  |  |  |  |  |  |  |
| Low | 2928 | 91 | 9853 | 9.2 | (7.5 to 11.3) | 1.00 | (Ref) | 1.00 | (Ref) |
| High | 1220 | 9 | 4186 | 2.1 | (1.1 to 4.1) | 0.43 | (0.21 to 0.86) | 0.46 | (0.23 to 0.92) |

EI, energy intake; PY, person-years; CI, confidence interval; HEI, high energy intake; HR, hazard ratio; HSC, high step counts; LEI, low energy intake; LSC, low step counts; Ref, reference; RERI, Relative Excess Risk due to Interaction; SC, step counts.

^a^ Model 1: Adjusted for age, sex, population density, and season of wear.

^b^ Model 2: Adjusted for Model 1 and body mass index, smoking status, alcohol consumption status, family structure, educational attainment, economic status, denture use, medication use, number of chronic diseases, and frailty status.

^c^ The additive interaction was calculated as the RERI using the following equation: RERI = (HR [HEI/HSC] –1) + (HR [HEI/LSC] + HR [LEI/HSC] –2). The values are shown as RERI (%). It is significant (p<0.05) if the 95% CI of the RERI is not above 0.

^d^ It is significant (*p*<0.05) if the 95% CI of the multiplicative interaction is not above 1.00.

**Supplementary Table 3.** Results of sensitivity analysis for the relationship between calibrated energy intake and daily step count on all-cause mortality using complete case data

|  | *n* | Event | PY | Event/1000 PY | | Model 1^a^ | | Model 2^b^ | |
| --- | --- | --- | --- | --- | --- | --- | --- | --- | --- |
|  |  |  |  | Rate | 95%CI | HR | 95%CI | HR | 95%CI |
| **EI×SC** |  | | | | | | | | |
| LEI/LSC | 928 | 47 | 3089 | 15.2 | (11.4 to 20.3) | 1.00 | (Ref) | 1.00 | (Ref) |
| HEI/LSC | 1151 | 18 | 3886 | 4.6 | (2.9 to 7.4) | 0.91 | (0.50 to 1.63) | 1.01 | (0.53 to 1.93) |
| LEI/HSC | 319 | 4 | 1087 | 3.7 | (1.4 to 9.8) | 0.40 | (0.14 to 1.13) | 0.45 | (0.16 to 1.27) |
| HEI/HSC | 562 | 1 | 1936 | 0.5 | (0.1 to 3.7) | 0.16 | (0.02 to 1.21) | 0.18 | (0.02 to 1.41) |
| *Interaction* |  |  |  |  |  |  |  |  |  |
| Additive^c^ |  |  |  |  |  | -0.15 | (-0.83 to 0.54) | -0.28 | (-1.75 to 1.20) |
| *p*-value |  |  |  |  | | 0.896 | | 0.763 | |
| Multiplicative^d^ |  |  |  | 0.46 | (0.05 to 4.41) | 0.44 | (0.05 to 4.22) | 0.41 | (0.04 to 3.92) |
| *p*-value |  |  |  | 0.502 | | 0.477 | | 0.436 | |
| **EI** |  |  |  |  |  |  |  |  |  |
| Low | 1247 | 51 | 4176 | 12.2 | (9.3 to 16.1) | 1.00 | (Ref) | 1.00 | (Ref) |
| High | 1713 | 19 | 5822 | 3.3 | (2.1 to 5.1) | 0.87 | (0.49 to 1.56) | 0.96 | (0.51 to 1.80) |
| **SC** |  |  |  |  |  |  |  |  |  |
| Low | 2079 | 65 | 6975 | 9.3 | (7.3 to 11.9) | 1.00 | (Ref) | 1.00 | (Ref) |
| High | 881 | 5 | 3023 | 1.7 | (0.7 to 4.0) | 0.32 | (0.13 to 0.82) | 0.35 | (0.14 to 0.88) |

CI, confidence interval; EI, energy intake; HEI, high energy intake; HR, hazard ratio; HSC, high step counts; LEI, low energy intake; LSC, low step counts; PY, person-years; Ref, reference; RERI, Relative Excess Risk due to Interaction; SC, step counts.

^a^ Model 1: Adjusted for age, sex, population density, and season of wear.

^b^ Model 2: Adjusted for Model 1 and body mass index, smoking status, alcohol consumption status, family structure, educational attainment, economic status, denture use, medication use, number of chronic diseases, and frailty status.

^c^ The additive interaction was calculated as the RERI using the following equation: RERI = (HR [HEI/HSC] –1) + (HR [HEI/LSC] + HR [LEI/HSC] –2). The values are shown as RERI (%). It is significant (p<0.05) if the 95% CI of the RERI is not above 0.

^d^ It is significant (*p*<0.05) if the 95% CI of the multiplicative interaction is not above 1.00.

**Supplementary Table 4**. Hazard ratios for calibrated energy intake per 100 step counts and all-cause mortality calculated using age- and sex-stratified multivariable Cox proportional hazards analysis

|  | *n* | Event | PY | Event/1000 PY | | Model 1^a^ | | Model 2^b^ | |
| --- | --- | --- | --- | --- | --- | --- | --- | --- | --- |
|  |  |  |  | Rate | 95%CI | HR | 95%CI | HR | 95%CI |
| ***Women*** |  |  |  |  |  |  |  |  |  |
| Q1 (30.0) | 506 | 6 | 1735 | 3.5 | (1.6 to 7.7) | 1.01 | (0.37 to 2.75) | 0.91 | (0.32 to 2.60) |
| Q2 (47.5) | 506 | 3 | 1713 | 1.8 | (0.6 to 5.4) | 0.41 | (0.12 to 1.45) | 0.34 | (0.09 to 1.26) |
| Q3 (67.8) | 507 | 12 | 1709 | 7.0 | (4.0 to 12.4) | 1.24 | (0.56 to 2.71) | 0.97 | (0.43 to 2.18) |
| Q4 (112.1) | 505 | 14 | 1697 | 8.2 | (4.9 to 13.9) | 1.00 | (Ref) | 1.00 | (Ref) |
| ***Men*** |  |  |  |  |  |  |  |  |  |
| Q1 (31.5) | 533 | 4 | 1825 | 2.2 | (0.8 to 5.8) | 0.24 | (0.08 to 0.68) | 0.28 | (0.10 to 0.81) |
| Q2 (50.5) | 533 | 7 | 1817 | 3.9 | (1.8 to 8.1) | 0.36 | (0.16 to 0.82) | 0.37 | (0.16 to 0.84) |
| Q3 (75.4) | 535 | 22 | 1805 | 12.2 | (8.0 to 18.5) | 0.84 | (0.49 to 1.42) | 0.91 | (0.53 to 1.57) |
| Q4 (143.9) | 534 | 43 | 1745 | 24.6 | (18.3 to 33.2) | 1.00 | (Ref) | 1.00 | (Ref) |
| *p for between groups* |  |  |  |  |  | 0.01 | | 0.325 | |
| ***<75 years*** |  |  |  |  |  |  |  |  |  |
| Q1 (29.3) | 724 | 1 | 2486 | 0.4 | (0.1 to 2.9) | 0.22 | (0.03 to 1.74) | 0.26 | (0.03 to 2.26) |
| Q2 (45.9) | 723 | 0 | 2463 | 0.0 | N/A | N/A | | N/A | |
| Q3 (65.4) | 720 | 3 | 2445 | 1.2 | (0.4 to 3.8) | 0.38 | (0.11 to 1.37) | 0.45 | (0.11 to 1.77) |
| Q4 (116.5) | 719 | 11 | 2424 | 4.5 | (2.5 to 8.2) | 1.00 | (Ref) | 1.00 | (Ref) |
| ***≥75 years*** |  |  |  |  |  |  |  |  |  |
| Q1 (35.5) | 319 | 13 | 1087 | 12.0 | (6.9 to 20.6) | 0.45 | (0.24 to 0.87) | 0.47 | (0.24 to 0.92) |
| Q2 (59.4) | 319 | 17 | 1065 | 16.0 | (9.9 to 25.7) | 0.58 | (0.32 to 1.05) | 0.57 | (0.31 to 1.03) |
| Q3 (87.4) | 318 | 26 | 1061 | 24.5 | (16.7 to 36.0) | 0.78 | (0.47 to 1.30) | 0.76 | (0.46 to 1.27) |
| Q4 (150.7) | 317 | 40 | 1015 | 39.4 | (28.9 to 53.7) | 1.00 | (Ref) | 1.00 | (Ref) |
| *p for between groups* |  |  |  |  |  | <0.001 | | <0.001 | |

CI, confidence interval; HR, hazard ratio; PY, person-years; Q, quartiles; Ref, reference. Q1 through Q4 include daily step counts of <39.4, 39.4–56.1, 56.2–80.9, and ≥81.0 kcal/100 steps, respectively, in women; <41.2, 41.2–61.5, 61.6–95.1, and ≥95.5 kcal/100 steps, respectively, in men; <38.4, 38.4–53.7, 53.8–78.9, and ≥79.0 kcal/100 steps, respectively, in individuals aged <75 years; and <47.4, 47.4–71.3, 71.4–106.1, and ≥106.2 kcal/100 steps, respectively, in individuals aged ≥75 years. The calibrated energy intake per 100 steps is expressed as quartiles (mean values).

^a^ Model 1: Adjusted for age, sex, population density, and season of wear.

^b^ Model 2: Adjusted for Model 1 and body mass index, smoking status, alcohol consumption status, family structure, educational attainment, economic status, denture use, medication use, number of chronic diseases, and frailty status.

**Supplementary Table 5**. Hazard ratios for z-score for calibrated energy intake and step counts and all-cause mortality calculated using age- and sex-stratified multivariable Cox proportional hazards analysis

|  | *n* | Event | PY | Event/1000 PY | | Model 1^a^ | | Model 2^b^ | |
| --- | --- | --- | --- | --- | --- | --- | --- | --- | --- |
|  |  |  |  | Rate | 95%CI | HR | 95%CI | HR | 95%CI |
| ***Women*** |  |  |  |  |  |  |  |  |  |
| Q1 (-1.85) | 504 | 25 | 1674 | 14.9 | (10.1 to 22.1) | 1.00 | (Ref) | 1.00 | (Ref) |
| Q2 (-0.58) | 509 | 4 | 1730 | 2.3 | (0.9 to 6.2) | 0.34 | (0.11 to 0.99) | 0.29 | (0.10 to 0.88) |
| Q3 (0.25) | 503 | 4 | 1715 | 2.3 | (0.9 to 6.2) | 0.56 | (0.18 to 1.79) | 0.49 | (0.15 to 1.60) |
| Q4 (1.68) | 508 | 2 | 1736 | 1.2 | (0.3 to 4.6) | 0.36 | (0.08 to 1.69) | 0.34 | (0.07 to 1.75) |
| ***Men*** |  |  |  |  |  |  |  |  |  |
| Q1 (-1.76) | 537 | 44 | 1766 | 24.9 | (18.5 to 33.5) | 1.00 | (Ref) | 1.00 | (Ref) |
| Q2 (-0.43) | 529 | 22 | 1788 | 12.3 | (8.1 to 18.7) | 1.05 | (0.61 to 1.80) | 1.11 | (0.63 to 1.96) |
| Q3 (0.51) | 536 | 7 | 1814 | 3.9 | (1.8 to 8.1) | 0.51 | (0.22 to 1.17) | 0.57 | (0.24 to 1.33) |
| Q4 (2.15) | 533 | 3 | 1824 | 1.6 | (0.5 to 5.1) | 0.31 | (0.09 to 1.08) | 0.32 | (0.09 to 1.18) |
| *p for between groups* |  |  |  |  |  | <0.001 | | 0.135 | |
| ***<75 years*** |  |  |  |  |  |  |  |  |  |
| Q1 (-1.01) | 721 | 8 | 2439 | 3.3 | (1.6 to 6.6) | 1.00 | (Ref) | 1.00 | (Ref) |
| Q2 (0.00) | 728 | 2 | 2464 | 0.8 | (0.2 to 3.2) | 0.25 | (0.05 to 1.19) | 0.30 | (0.06 to 1.48) |
| Q3 (0.78) | 720 | 5 | 2454 | 2.0 | (0.8 to 4.9) | 0.78 | (0.25 to 2.40) | 0.74 | (0.22 to 2.46) |
| Q4 (2.23) | 717 | 0 | 2462 | 0.0 | N/A | N/A | | N/A | |
| ***≥75 years*** |  |  |  |  |  |  |  |  |  |
| Q1 (-2.51) | 318 | 27 | 1048 | 25.8 | (17.7 to 37.6) | 1.00 | (Ref) | 1.00 | (Ref) |
| Q2 (-1.58) | 319 | 29 | 1042 | 27.8 | (19.3 to 40.0) | 1.11 | (0.66 to 1.87) | 1.27 | (0.74 to 2.19) |
| Q3 (-0.88) | 317 | 20 | 1063 | 18.8 | (12.1 to 29.2) | 0.87 | (0.48 to 1.55) | 0.95 | (0.52 to 1.74) |
| Q4 (0.47) | 319 | 20 | 1074 | 18.6 | (12.0 to 28.9) | 0.84 | (0.47 to 1.52) | 0.98 | (0.51 to 1.86) |
| *p for between groups* |  |  |  |  |  | <0.001 | | <0.001 | |

CI, confidence interval; HR, hazard ratio; PY, person-years; Q, quartiles; Ref, reference. Q1 through Q4 include z-score for calibrated energy intake and step counts of <-1.06, -1.06–-0.16, -0.15–0.72, and ≥0.73 scores, respectively, in women; <-0.93, -0.92–0.04, 0.05–1.06, and ≥1.07 scores, respectively, in men; <0.38, 0.38–0.36, 0.37–1.27, and ≥1.28 scores, respectively, in individuals aged <75 years; and <-1.92, -1.92–-1.25, -1.24–-0.48, and ≥-0.47 scores, respectively, in individuals aged ≥75 years. The z-scores are expressed as quartiles (mean values).

^a^ Model 1: Adjusted for age, sex, population density, and season of wear.

^b^ Model 2: Adjusted for Model 1 and body mass index, smoking status, alcohol consumption status, family structure, educational attainment, economic status, denture use, medication use, number of chronic diseases, and frailty status.


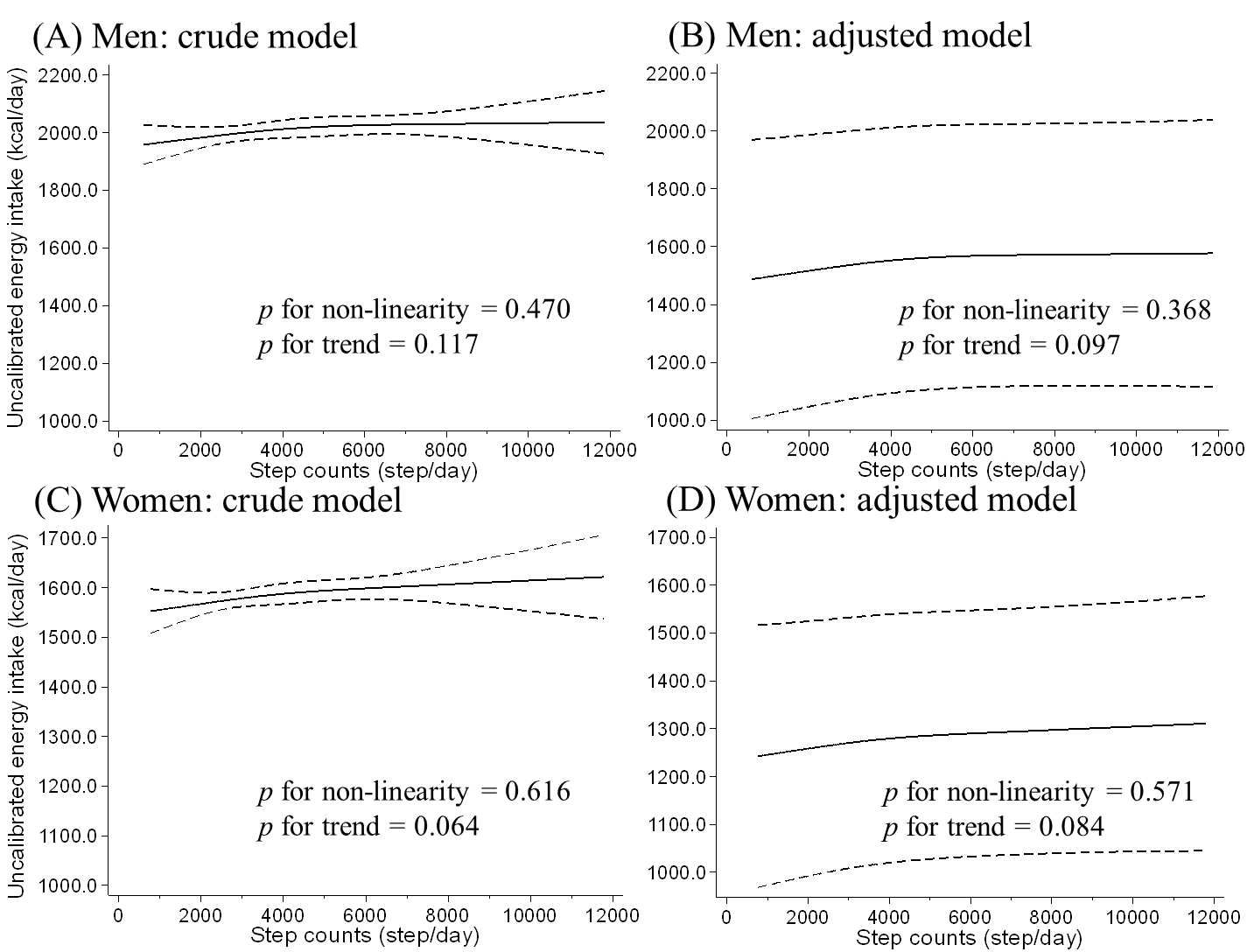


**Supplemental Figure 1**. Relationship between uncalibrated energy intake and daily step counts by a restricted cubic spline model among older adults.

[A] crude model and [B] multivariable adjusted model in 2,107 men and [C] crude model and [D] multivariable adjusted model in 2,011 women. Because the data were sparse, we truncated the analysis at 12,000 steps/day (99% of the distribution). Solid lines represent mean calibrated energy intake, dashed lines represent 95% confidence intervals (CI). The adjustment factors were age, population density, season of wear, body mass index, smoking status, alcohol consumption status, family structure, educational attainment, economic status, denture use, medication use, number of chronic diseases, and frailty status.
